# Personalized loading conditions for homogenized finite element analysis of the distal sections of the radius

**DOI:** 10.1007/s10237-022-01656-4

**Published:** 2022-12-07

**Authors:** Denis Schenk, Philippe Zysset

**Affiliations:** ARTORG Center for Biomedical Engineering Research, University of Bern, Bern, Switzerland

**Keywords:** Boundary conditions, Distal radius, Finite element analysis, Homogenized, Personalized

## Abstract

The microstructure of trabecular bone is known to adapt its morphology in response to mechanical loads for achieving a biomechanical homeostasis. Based on this form–function relationship, previous investigators either simulated the remodeling of bone to predict the resulting density and architecture for a specific loading or retraced physiological loading conditions from local density and architecture. The latter inverse approach includes quantifying bone morphology using computed tomography and calculating the relative importance of selected load cases by minimizing the fluctuation of a tissue loading level metric. Along this concept, the present study aims at identifying an optimal, personalized, multiaxial load case at the distal section of the human radius using in vivo HR-pQCT-based isotropic, homogenized finite element (hFE) analysis. The dataset consisted of HR-pQCT reconstructions of the 20 mm most distal section of 21 human fresh-frozen radii. We simulated six different unit canonical load cases (FX palmar–dorsal force, FY ulnar–radial force, FZ distal–proximal force, MX moment about palmar–dorsal, MY moment about ulnar–radial, MZ moment about distal–proximal) using a simplified and efficient hFE method based on a single isotropic bone phase. Once we used a homogeneous mean density (shape model) and once the original heterogeneous density distribution (shape + density model). Using an analytical formulation, we minimized the deviation of the resulting strain tensors **ε**(**x**) to a hydrostatic compressive reference strain **ε**_0_, once for the 6 degrees of freedom (DOF) optimal (OPT) load case and for all individual 1 DOF load cases (FX, FY, FZ, MX, MY, MZ). All seven load cases were then extended in the nonlinear regime using the scaled displacements of the linear load cases as loading boundary conditions (MAX). We then compared the load cases and models for their objective function (OF) values, the stored energies and their ultimate strength using a specific torsor norm. Both shape and shape + density linear-optimized OPT models were dominated by a positive force in the *z*-direction (FZ). Transversal force DOFs were close to zero and mean moment DOFs were different depending on the model type. The inclusion of density distribution increased the influence and changed direction of MX and MY, while MZ was small in both models. The OPT load case had 12–15% lower objective function (OF) values than the FZ load case, depending on the model. Stored energies at the optimum were consistently 142–178% higher for the OPT load case than for the FZ load case. Differences in the nonlinear response maximum torsor norm ‖t‖ were heterogeneous, but consistently higher for OPT_MAX than FZ_MAX. We presented the proof of concept of an optimization procedure to estimate patient-specific loading conditions for hFE methods. In contrast to similar models, we included canonical load cases in all six DOFs and used a strain metric that favors hydrostatic compression. Based on a biomechanical analysis of the distal joint surfaces at the radius, the estimated load directions are plausible. For our dataset, the resulting OPT load case is close to the standard axial compression boundary conditions, usually used in HR-pQCT-based FE analysis today. But even using the present simplified hFE model, the optimized linear six DOF load case achieves a more homogeneous tissue loading and can absorb more than twice the energy than the standard uniaxial load case. The ultimate strength calculated with a torsor norm was consistently higher for the 6-DOF nonlinear model (OPT_MAX) than for the 1-DOF nonlinear uniaxial model (FZ_MAX). Defining patient-specific boundary conditions may decrease angulation errors during CT measurements and improve repeatability as well as reproducibility of bone stiffness and strength estimated by HR-pQCT-based hFE analysis. These results encourage the extension of the present method to anisotropic hFE models and their application to repeatability data sets to test the hypothesis of reduced angulation errors during measurement.

## Introduction

Advanced high-resolution in vivo imaging techniques such as high-resolution peripheral quantitative computed tomography (HR-pQCT) allow to capture and characterize in detail trabecular and cortical bone morphology at the distal radius and tibia. The application of micro-finite element (μFE) analysis on such images is a powerful research tool to perform mechanical analysis of 9–10 mm bone sections, and computed fracture load proved to be the best predictor of incident fracture risk in older women (Samelson et al. 2019). It is evident and well established that a specific bone fracture risk correlates best with the strength of the specific anatomical site under the specific loading mechanism of the injury. The critical zone of fractures such as Colle's fracture in the distal segments of the radius extends beyond 9–10 mm HR-pQCT sections, and multiple bone sections appear to be necessary for proper assessment of distal bone strength (Varga et al. 2010; Baumbach et al. 2011; Mueller et al. 2011). Against this trend toward evaluating multiple sections, a short processing time is essential for the potential clinical application of such computational methods. Despite the continuous improvement of computational power, the necessary resources for μFE of a 9–10 mm bone section remain high. Therefore, so-called homogenized finite element (hFE) methods seem to be an attractive alternative to μFE models. They are based on element orders of magnitude larger than the resolution of the underlying HR-pQCT reconstructions and take a fraction of the time and computational resources to solve. These hFE models offer similar quality of linear relationship to experimental measures (*R*^2^ > 0.9) (Hosseini et al. 2017a; Arias-Moreno et al. 2019) and similar or improved repeatability in multiple-section HR-pQCT measurements at the distal sections of radius and tibia (Schenk et al. 2020). Furthermore, we recently established a reference database for bone stiffness and strength of multiple-section reconstructions of the young and healthy Swiss population (Stuck et al. 2020).

Beyond the sections of interest to compute fracture loads, the detailed personalized morphology included in the HR-pQCT reconstructions of distal bone sections at the radius and tibia contrasts with the standard axial compression usually applied by both μFE and hFE models. Although several studies suggest that axial compression is the primary loading mode (Varga et al. 2010, 2011; Christen et al. 2013a), the distal joint surfaces transfer heterogeneous stress amplitudes in multiple directions under physiological loading or in case of an accident, such as falling on the outstretched hand. The influence of these boundary conditions is currently not accounted for in clinical studies exploiting HR-pQCT for assessing bone strength at the radius and tibia.

Human bone is a heterogeneous composite continuously remodeled and adapts to changing environments by highly regulated processes, initiated among other endocrine influences by mechanical stimuli. Evidence suggests that the sensitivity of bone tissue to mechanical stimuli and consequently the bone tissue’s ability to adapt to external mechanical environments is based on specialized cells called osteocytes. Together with lining cells and a network of canaliculi, they can transmit stimuli to surfaces with high strains and induce bone formation until strains are normalized (Burger and Klein-Nulend 1999). The computational implementation of this functional adaption theory was pioneered by Carter and Beaupré (2001), Beaupré et al. (1990), Cowin and Hegedus (1976), Hegedus and Cowin (1976), and later investigated by many others and transitioned to the microstructural level by Huiskes et al. (2000).

If the physiological loading environment influences the trabecular bone microstructure, the respective shape, density distribution, and orientation in space must inversely contain information about the loading conditions that formed the respective microstructure. Or in other words, the loading conditions that the respective microstructure was optimized to sustain. Accordingly, an inverse relationship exists between bone morphology and the physiological loading history, the respective bone morphology was optimized for. Fischer and colleagues used an optimization procedure to determine a set of plausible loads for a given density distribution using artificial 2D models of bone ends (Fischer et al. 1995) and, later, the proximal femur (Fischer et al. 1996).

Christen and colleagues brought the above-described inverse optimization problem of back-computing a mechanical loading history based on bone architecture to a microstructural level. They first applied μFE analysis on murine bone remodeling models to minimize the variance of strain energy density (Christen et al. 2012). Later, their load estimation algorithm was validated on synthetic bone micro-architectures (Christen et al. 2013b) and applied to high-resolution peripheral quantitative computed tomography (HR-pQCT) reconstructions of the distal 9–10 mm segment of radii (Christen et al. 2016).

Estimating personalized loading boundary conditions by optimization procedures requires multiple model evaluations and increases processing time. A transition back to continuum models using established hFE analysis methods would be beneficial. Accordingly, this study is a proof of concept and aims to identify a personalized and multi-axial load case using simplified, isotropic hFE models from clinical HR-pQCT reconstructions of distal double-sections of the human radius. The estimation of the personalized loading boundary conditions or loading history is based on an optimization procedure using a strain metric that is not symmetric in compression and tension. Further, we were interested in the distinct influence of shape and density distribution on the estimated personalized loading history. Accordingly, we designed and applied optimization procedures on hFE models mapping the heterogeneous density distribution and on models containing an artificial, unified, homogeneous mean density.

## Material and methods

**Fig. 1:**
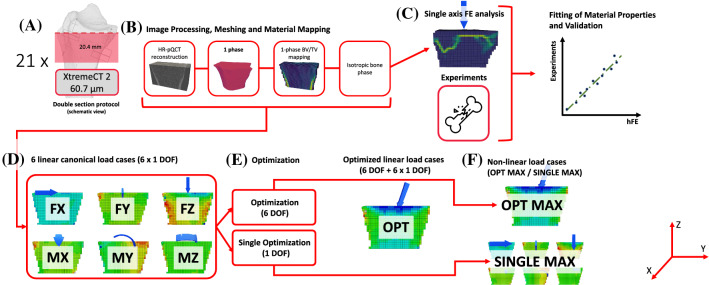
Graphical abstract summarizing the material and methods section. **A, B** **+ Attachment: homogenized finite element method:** **A** Measurement acquisition using a second-generation HR-pQCT scanner (XtremeCT2) at an isotropic voxel size of 60.7 μm and schematic representation of the double section scan regions, **B** Overview of simplified hFE analysis method based on a single bone phase modeled as elastic–plastic constitutive material including damage, acc. to Schwiedrzik and Zysset (2013, Attachment) Fitting of material properties and experimental validation of hFE analysis method using experimental results from Hosseini et al. (2017a). **C–F** **Personalizing loading boundary conditions: C** Simulation of six linear canonical load cases with unit load boundary conditions along the 6-DOF (FX, FY, FZ, MX, MY, MZ), **D** 6-DOF (OPT) and 1-DOF (FX, FY, FZ, MX, MY, MZ) optimization procedure, **E** simulation of linear 6-DOF OPT load case and linear single 1-DOF optimized load cases, **F** Simulation end evaluation of nonlinear MAX load cases using distributor of respective linear load cases as displacement-controlled loading boundary conditions

A summary of the applied methods is given in Fig. 1A, B. First, we created hFE models based on HR-pQCT reconstructions of the distal part of 21 human radii, with a mean homogeneous density distribution (shape model) and with the measured heterogeneous density distribution (shape + density model). (C) For both models, we evaluated 6 linear canonical load cases in all 6 DOF using unit loading boundary conditions (1 N, 1Nm). (D, E) Then, we applied an optimization procedure to estimate an optimized load case by minimizing strain deviations with reference to a hydrostatic compressive reference strain, once in 6 DOF (OPT) and each for the individual 1 DOF load cases (FX–FZ). (F) The seven resulting load cases were then extended in the nonlinear regime using scaled displacements of the linear load cases as loading boundary conditions (MAX).

### Sample preparation, measurement, image processing and compression test

Hosseini and colleagues (Baumbach et al. 2011) established experimental compression data, and HR-pQCT measurements were established in a previous study. In brief, the dataset consists of 21 fresh frozen distal segments of human radii from 6 females (79.8 y [70y, 92y]) and 8 males (72.3y [59y, 87y]). All forearm samples were measured on a second-generation HR-pQCT scanner (XtremeCT2, Scanco Medical AG, Brütisellen, Switzerland) at an isotropic voxel size of 60.7 μm and using the standard clinical procedure. The 20.4-mm segments are located 5 mm from the lowest part of the distal subchondral plate and include the adjacent proximal two HR-pQCT acquisition sections (double section, 2 stacks of 168 slices, 20.4 mm).

### Homogenized finite element analysis method

The hFE method used in the current project was adapted and simplified from earlier studies (Arias-Moreno et al. 2019; Hosseini et al. 2017b) and validated using the above-described set of distal segments of radii and experimental results. An overview of the analysis method and its experimental validation is provided in the attachment. All subsequent evaluations were performed on two types of models: (1) with the original heterogeneous BV/TV distribution of the HR-pQCT reconstructions (shape + density) and (2) with a homogeneous mean BV/TV distribution equal to the mean BV/TV of the masked HR-pQCT reconstruction (shape).

### Personalized optimization of loading direction

Without a decomposition in time, an average physiological loading history of a bone segment without attached tendons and ligaments, located at some distance from the joint, can be represented by a linear combination of elementary torsors applied to a rigid plane or point:

1 $$\left\{\mathbf{f}, \mathbf{m}\right\}= \sum_{i=1}^{6}{\alpha }_{i}{\left\{\mathbf{f}, \mathbf{m}\right\}}_{i}$$

With $\mathbf{f}$ and $\mathbf{m}$ being resultant 3D forces and moments describing the loading history and ${\alpha }_{i}$ the linear weights of the combination of elementary torsors, that are real and not necessarily positive to distinguish both tension and compression.

Since we evaluate linear elastic models (i.e., linear boundary value problem) in the optimization process, the solution for displacement and rotation is linear. Therefore, a stiffness tensor ${\mathbb{K}}$ exists that relates a descriptor $$\left[\begin{array}{c}{\varvec{u}}\\{\varvec{\theta}}\end{array}\right]$$ to the torsor $$\left[\begin{array}{c}{\varvec{f}}\\ {\varvec{m}}\end{array}\right]$$:

2 $$\left[\begin{array}{c}{\varvec{u}}\\{\varvec{\theta}}\end{array}\right]={\left[\begin{array}{cc}{{\varvec{S}}}_{fu}& {{\varvec{S}}}_{f\theta }\\ {{\varvec{S}}}_{mu}& {{\varvec{S}}}_{m\theta }\end{array}\right]}^{-1}\left[\begin{array}{c}{\varvec{f}}\\ {\varvec{m}}\end{array}\right]$$

With ${\varvec{u}}$ and ${\varvec{\theta}}$ being the displacements [mm] and the angles of rotation [rad], ${\varvec{f}}$ and ${\varvec{m}}$ the force and moment vector and ${\mathbb{K}}^{-1}$ the relating second-order tensor that can be subdivided into 2 × 2 sub-matrices of dimension 3 × 3 that have unique physical units and properties. Linearity holds for the displacement gradient ${\varvec{u}}$; thus, it holds as well for infinitesimal strain ${\varvec{\varepsilon}}$ and stress ${\varvec{\upsigma}}$. Accordingly, strains of an arbitrary torsor can be described as:

3 $${\varvec{\upvarepsilon}}\left({\varvec{x}}\right)={\varvec{\upvarepsilon}}\left({\varvec{x}};{\alpha }_{i}\{{\varvec{f}},{\varvec{m}}{\}}_{i}\right)={\sum }_{i=1}^{6}{\alpha }_{i}{{\varvec{\upvarepsilon}}}_{i}\left({\varvec{x}}\right)$$

**Fig. 2:**
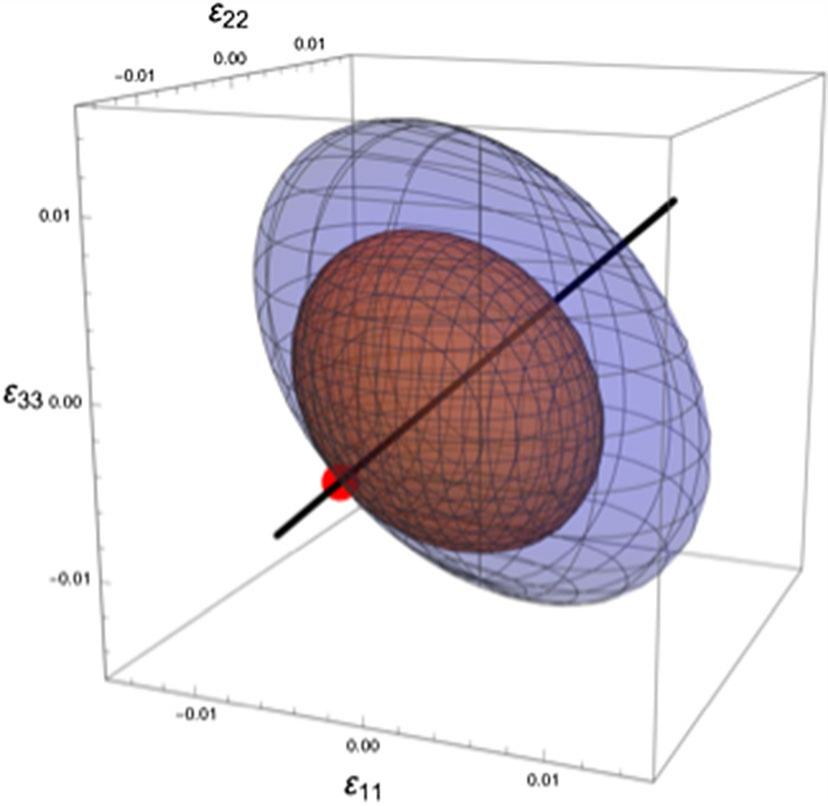
Graphical rational for a compressive hydrostatic reference strain tensor, showing an isotropic material orientation (black line), a quadric isotropic damage surface (red ellipsoid) and a corresponding constant strain energy density contour (blue ellipsoid). Maximizing the strain energy density under the constraint of not exceeding a quadric isotropic damage criterion results in a single optimum (red dot) of a hydrostatic compressive reference strain

In contrast to classical strain or equivalent strain norms, the strain tensor distinguishes tensile from compressive strains. In the case of an isotropic material description, the compliance tensor is generally assumed isotropic, and under the constraint of not exceeding a quadric isotropic damage criterion, a hydrostatic compressive strain (${-\varepsilon }_{0}{\varvec{I}}$) leads to the maximum strain energy density (or the minimal complementary energy density) (Fig. 2).

Accordingly, we formulated an optimization problem consisting of finding a linear combination of ${\alpha }_{i}$ of the canonical torsors that minimize the variance of the strain tensor ${\varvec{\varepsilon}}\left({\varvec{x}}\right)$ to an isotropic and volume-fraction-dependent constant reference compressive strain tensor ${-\varepsilon }_{0}{\varvec{I}}$**,** over a relevant bone domain $\Omega$.

4 $${Min}_{{\alpha }_{i}}OF\left({\alpha }_{i}\right)={Min}_{{\alpha }_{i}}{\int }_{\Omega }\left({\varvec{\varepsilon}}\left({\varvec{x}}\right){\rho }^{r}+{\varepsilon }_{0}{\varvec{I}}\right):\left({\varvec{\varepsilon}}\left({\varvec{x}}\right){\rho }^{r}+{\varepsilon }_{0}{\varvec{I}}\right)dV$$

With $OF$ being the objective function, ${\varvec{\varepsilon}}\left({\varvec{x}}\right)$ the strain tensor of the general load at position ${\varvec{x}}$ in the domain $\Omega$, ${-\varepsilon }_{0}{\varvec{I}}$ a constant hydrostatic compressive reference tensor, $\rho$ the bone volume fraction BV/TV, and $r$ the exponent of the volume fraction dependency. Since ${\alpha }_{i}$ are not bound, ${\varepsilon }_{0}$ will determine their scaling factor and was defined as ${\varepsilon }_{0}=0.1\%$*.*

Using (4) and by replacing the integral over the volume by a sum over all FE elements (voxels are of equal volume), the objective function becomes

5 $$\frac{1}{{N}_{elem}}\sum_{n=1}^{{N}_{elem}}\left(\sum_{i=1}^{6}\sum_{j=1}^{6}{\alpha }_{i}{\alpha }_{j}{}^{n}{\rho }^{2r}{}^{n}{\varepsilon }_{i}:{}^{n}{\varepsilon }_{j}+2{\varepsilon }_{0}\sum_{j=1}^{6}{\alpha }_{j}{}^{n}{\rho }^{r}Tr({}^{n}{\varepsilon }_{j})+3{\varepsilon }_{0}^{2}\right)$$

The derivative to $\alpha$ defining the stationary point is

6 $$2\sum_{j=1}^{6}{\alpha }_{j}\frac{1}{{N}_{elem}}\sum_{n=1}^{{N}_{elem}}{}^{n}{\rho }^{2r}{}^{n}{\varepsilon }_{i}:{}^{n}{\varepsilon }_{j}+2{\varepsilon }_{0}\frac{1}{{N}_{elem}}\sum_{j=1}^{6}{}^{n}{\rho }^{r}Tr\left({}^{n}{\varepsilon }_{j}\right)=0$$

which generates a linear problem

7 $${A}_{ij}{\alpha }_{j}+b=0\quad i=1,6$$

With

8 $${A}_{ij}=2\frac{1}{{N}_{elem}}\sum_{n=1}^{{N}_{elem}}{}^{n}{\rho }^{2r}{}^{n}{\varepsilon }_{i}:{}^{n}{\varepsilon }_{j}\quad b=2{\varepsilon }_{0}\frac{1}{{N}_{elem}}\sum_{j=1}^{6}{}^{n}{\rho }^{r}Tr\left({}^{n}{\varepsilon }_{j}\right)$$

#### Volume fraction dependency parameter *r*

We performed a preliminary sensitivity analysis for the volume fraction dependency parameter $r$. First, an optimized set of ${\alpha }_{i}$ was computed for each distal double section of the radii dataset using $r=0$. Then, $r$ was iterated with a step size of $+0.001$, and $OF$ value was computed for each iteration. The resulting minimum values $r\left(Min\left(OF\right)\right)$ were averaged over all radius samples and used for subsequent optimizations.

#### Linear optimized load case OPT

For each radius sample, we evaluated six linear unit load cases (canonical load cases) (FX, FY, FZ, MX, MY, MZ) using the above-described hFE analysis procedure. The displacements of all nodes of the most proximal surface of all six models were fixed in 6 DOFs ($$\left[\begin{array}{c}{\varvec{u}}\\{\varvec{\theta}}\end{array}\right] = \left[\begin{array}{c}0\\ 0\end{array}\right] )$$. The nodes at the most distal surface were kinematically coupled to a virtual reference node positioned at the distal surface along the central vertical axis of the bone segment volume. For each load case, the virtual reference node was loaded with a unit load (force load cases: 1 N, moment load cases: 1 Nm) in one of the 6 DOFs. The remaining 5 DOFs were fixed to zero displacements and rotations. During simulation, the following parameters were recorded: the six-dimensional displacement and rotation vector $\mathbf{d}$ of the virtual reference node, the resulting strain tensors${\varvec{\varepsilon}}\left({\varvec{x}}\right)$, and density $\rho$ of all FE elements. Using formulas (7) and (8) and the previously established volume fraction dependency parameter $r$ the weights ${\alpha }_{i}$ of the linear combination of canonical load cases at the minimum of the optimization function were computed. The resulting combination of all six ${\alpha }_{i}$ defines the boundary conditions of the optimized linear load case (OPT).

The present dataset of distal double-sections of radii includes both left and right forearm samples. The respective ${\alpha }_{i}$ have a different orientation to the radius anatomy. Accordingly, we normalized orientation in the FE models according to anatomical landmarks (${\alpha }_{FX}$, ${\alpha }_{MY}$ and ${\alpha }_{MZ}$ were changed sign).

#### Single linear load case optimization (FX, FY, FZ, MX, MY, MZ)

The above-described optimization procedure was reduced to a single DOF to compare the optimized load case OPT to the individual load cases FX, FY, FZ, MX, MY, and MZ in the linear material regime. All ${\alpha }_{i}$ were forced to zero, except the one corresponding to the respective load case. The system of initially six linear equations in formula (8) is reduced to a single linear equation:

9 $${\alpha }_{i}=\frac{{-b}_{i}}{{A}_{ii}}=\frac{-2{\varepsilon }_{0}\frac{1}{{N}_{elem}}\sum_{j=1}^{6}{}^{n}{\rho }^{r}Tr\left({}^{n}{\varepsilon }_{j}\right)}{2\frac{1}{{N}_{elem}}\sum_{n=1}^{{N}_{elem}}{}^{n}{\rho }^{2r}{}^{n}{\varepsilon }_{i}:{}^{n}{\varepsilon }_{i}}\quad i=1,6$$

#### Linear energy at optimum

For the resulting seven linear load cases (OPT, FX, FY, FZ, MX, MY, MZ), we computed an optimized set of ${\alpha }_{i}$ based on formulas (8) and (9) and the respective value of the objective function according to formula (5). To compare the different linear load cases, we evaluated the stored elastic energy at the optimum as the mechanical work performed on the virtual reference node or the dot product between torsor ${\varvec{t}}$ and descriptor ${\varvec{d}}$:

10 $${W}_{\rm opt}={\left[\begin{array}{c}{\varvec{f}}\\ {\varvec{m}}\end{array}\right]}_{\rm opt}\cdot {\left[\begin{array}{c}{\varvec{u}}\\{\varvec{\theta}}\end{array}\right]}_{\rm opt}\quad {W}_{\rm single}={\alpha }_{i}^{2}\left({\left[\begin{array}{c}{\varvec{f}}\\ {\varvec{m}}\end{array}\right]}_{i}\cdot {\left[\begin{array}{c}{\varvec{u}}\\{\varvec{\theta}}\end{array}\right]}_{i}\right)$$

#### Nonlinear analyses

The linear hFE models were loaded using force and moment boundary conditions. For nonlinear analyses evaluating a maximum force or moment (ultimate load evaluation), loading boundary conditions must be displacement controlled. Therefore, we used the descriptor ${\varvec{d}}$ of the respective previous linear analyses as loading boundary conditions for the following nonlinear analyses. To reach a maximum in the nonlinear regime, the descriptors were linearly scaled.

Comparing the nonlinear (MAX) load cases is not straightforward. The reference value for comparing linear load cases was the minimum value of the optimization function $Min\left(OF\right)$. However, in nonlinear analyses, such an optimum is not defined anymore. Furthermore, a measure of maximum fracture load, such as for uni-axial load cases, is missing. To compare the nonlinear load cases to each other, we therefore introduce the concept of a torsor and descriptor norm. We build a stiffness tensor ${\mathbb{K}}$ from the six linear load cases, representing the linear material response in all 6 DOF according to formula 2 and compute a descriptor and torsor norm as follows:

11 $$\Vert d\Vert =\sqrt{{\varvec{d}}\cdot {\mathbb{K}}{\varvec{d}}}$$

12 $$\Vert t\Vert =\sqrt{{\varvec{t}}\cdot {\mathbb{K}}^{-1}{\varvec{t}}}$$

The norms both have the physical unit of $\sqrt{Nmm}$. An exemplary torsor–descriptor norm curve is shown in Fig. 7. The respective maximum of the torsor norm can be used as a measure to compare the nonlinear response of multi-axial load cases.

### Statistics

Differences in linear energy and OF value between linear OPT and FZ load, and between shape and shape + density model, were tested with a paired one-sided nonparametric Mann–Whitney–Wilcoxon test after confirming non-normality of the variables with Shapiro–Wilk tests. The same procedure was applied to test the difference in torsor norm between the nonlinear OPT_MAX and FZ_MAX load case and between the shape and the shape + density model of the nonlinear OPT_MAX and FZ_MAX load case.

All statistical analyses were performed in R (The R foundation for statistical computing, Austria, version 3.6.3), and the level of statistical significance was set to *p* < 0.05.

## Results

The objective function value was minimized for a mean volume fraction dependency parameter $r$ of 0.008 (sd: 0.006, range: [0.0005, 0.023]). All subsequent optimization evaluations were generated using this value for $r$. The latter presented results are all summarized in Table 1 as means, standard deviations and ranges for the OPT, OPT_MAX, FZ and FZ_MAX load case.

**Table 1:** Summary of comparison between linear OPT and FZ and nonlinear OPT_MAX and FZ_MAX load cases, differentiated by model type (shape and shape + density). SD = standard deviation, OF value = objective function value according to formula (5), linear energy at optimum according to formula (10), Maximum $\Vert t\Vert$ = maximum torsor norm according to formula (12). A (*) indicates a significant paired on-sided nonparametric Mann–Whitney–Wilcoxon test on the differences between OPT and FZ or OPT_MAX and FZ_MAX. A (#) indicates a significant paired one-sided nonparametric Mann–Whitney–Wilcoxon test on the differences between shape + density and density model of OPT load case and a ( +) indicates the same test on FZ or FZ_MAX

	Shape		Shape + density	
	Mean ± SD [min, max]		Mean ± SD [min, max]	
**Linear analyses**	**OPT**	**FZ**	**OPT**	**FZ**
OF value [${10}^{-6}$]	2.699 ± 0.021 [2.672, 2.744]	2.727 ± 0.044 [2.681, 2.832]	2.653 ± 0.037 [2.615, 2.762]	2.700 ± 0.049 [2.624, 2.815]
*Comparison*	*#	+	*#	+
Linear energy at optimum [$Nmm$]	3.992 ± 2.473 [1.227, 9.173]	1.823 ± 1.231 [0.483, 4.440]	4.395 ± 2.900 [1.268, 10.868]	1.863 ± 1.454 [0.464, 5.294]
*Comparison*	*#		*#	
**Nonlinear analyses**	**OPT_MAX**	**FZ_MAX**	**OPT_MAX**	**FZ_MAX**
Maximum $\Vert t\Vert$ [$\sqrt{Nmm}$]	25.145 ± 8.340 [14.743, 38.788]	24.083 ± 8.616 [13.022, 37.295]	28.193 ± 9.928 [14.154, 46.404]	26.953 ± 9.924 [13.586, 46.292]
*Comparison*	*#	+	*#	+

### Linear load analyses

**Fig. 3:**
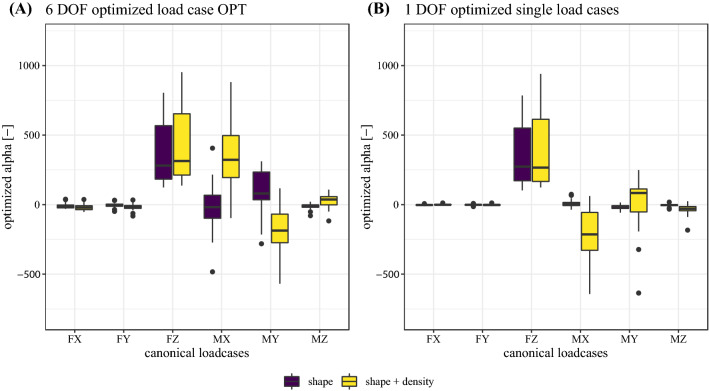
Linear weights ${\alpha }_{i}$ of **A** the OPT and **B** of the single optimized linear load cases, differentiated by the model type (shape or shape + density)

The resulting linear weights ${\alpha }_{i}$ of the optimization procedure are shown in Fig. 3A, B for the load case OPT and the six single optimized load cases (FX–MZ), respectively. In both models (shape and shape + density), the mean force DOFs are dominated by a positive force in the *z*-direction (FZ). The transversal force DOFs (FX and FY) are close to zero. The mean moment DOFs are different for both models. In the shape model, the largest mean moment DOF is a positive moment around the y-axis (MY) and a medium positive or negative moment around the *x*-axis (MX). However, in the shape + density model, mean moment DOFs are dominated by a positive moment around the *x*-axis (MX) and a medium negative moment around the *y*-axis (MY). Mean moments around the *z*-axis (MZ) are small for both models but higher in the shape + density model. Optimizing the six single linear load cases resulted in the largest ${\alpha }_{i}$ for the FZ load case for both models. In the shape model, the ${\alpha }_{i}$ of all other load cases were comparably small. However, in the shape + density model a medium negative ${\alpha }_{i}$ for MY load case and medium positive or negative ${\alpha }_{i}$ for MZ load case are present.

**Fig. 4:**
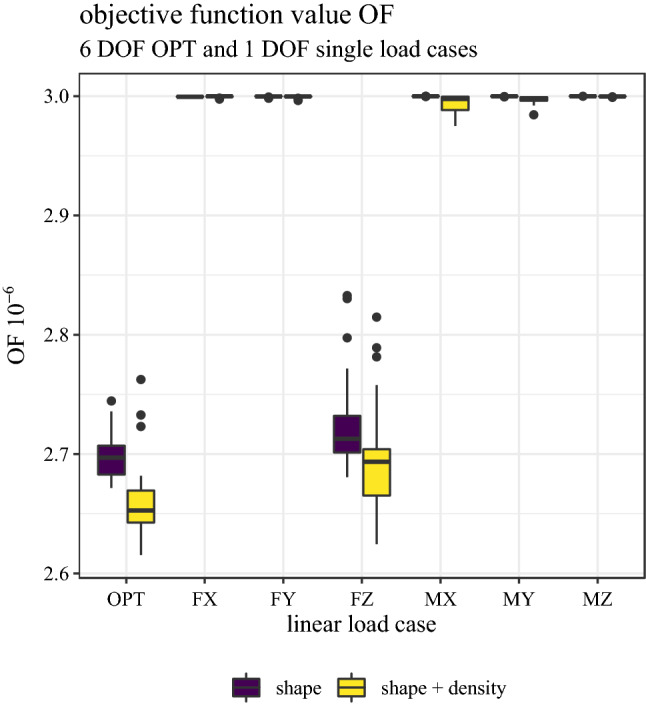
Value of objective function at respective optimized minimum for the linear optimized load case OPT and the six linear optimized single load cases (FX, FY, FZ, MX, MY, MZ). All load cases are differentiated by the respective model (shape and shape + density)

The corresponding objective function values are shown in Fig. 4A. The linear OPT load case resulted in significantly lower OF values on average than the FZ load case in both models (shape: *p* > 0.001, shape + density: *p* < 0.001). Relative differences in OF values between OPT and FZ load case were −1.0% ± 0.9% and −1.4% ± 0.8% for shape and shape + density model, respectively. Associated ranges were [−0.2%, −3.84%] and [−0.3%, −2.9%]. All other load cases resulted in OF values close to the maximum of 3.0, with OF values of the shape + density of the linear load cases MX and MY being slightly lower. Differences between OF values of the shape and the shape + density were significant for both load cases (OPT: *p* < 0.001, FZ: *p* < 0.001).

**Fig. 5:**
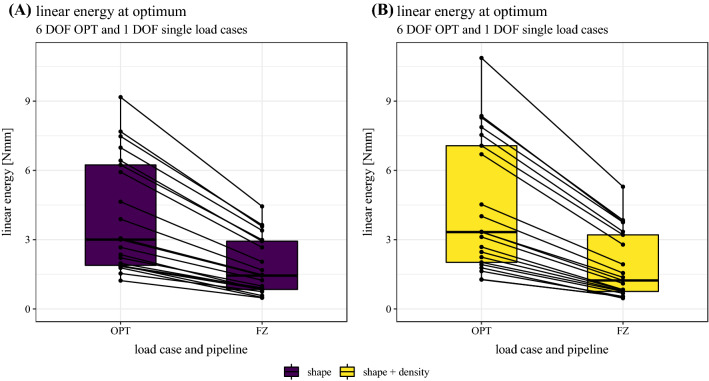
Linear weights ${\alpha }_{i}$ of **A** the optimized linear load case OPT and **B** of the six single optimized linear load cases (FX, FY, FZ, MX, MY, MZ). Both are differentiated by either the model type (shape or shape + density)

Finally, linear energies at optimum for the OPT and FZ load case are presented in Fig. 5. All other single linear load cases (FX, FY, MX, MY, MZ) resulted in linear energies close to zero. Linear energy of the OPT load case was consistently and significantly higher than of the FZ load case in both models (shape: *p* < 0.001, shape + density: *p* < 0.001). Mean relative differences in linear energies between OPT and FZ load case were 132.1% ± 41.7% and 159.6% ± 44.2% for the shape and shape + density model, respectively. Associated ranges were [104.7%, 256.5%] and [105.3%, 283.9%]. Differences between the shape and the shape + density model were only significant for the OPT load case (*p* < 0.001), but not for the FZ load case (*p* = 0.489).

All above-presented results of the linear analyses are summarized in Table 1.

### Nonlinear analyses

**Fig. 6:**
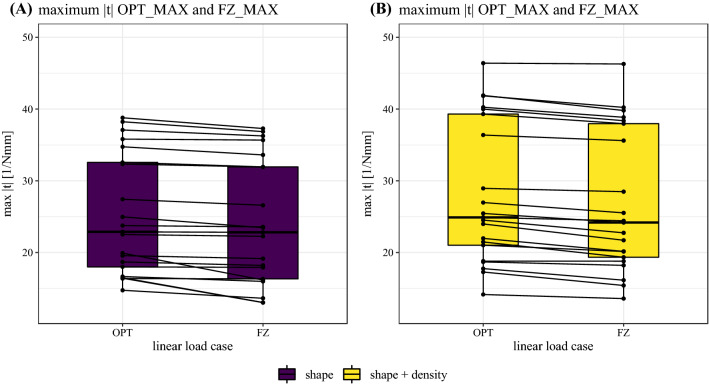
The maximum torsor norm $\Vert t\Vert$ according to formula (12) for **A** shape and **B** shape + density models. Both figures differentiate nonlinear load case OPT_MAX and FZ_MAX. The lines connect data points originating from the same samples

The maximum torsor norm $\Vert t\Vert$ of the OPT_MAX and FZ_MAX load case is shown in Fig. 6(A: shape, B: shape + density). Lines connect corresponding samples of the OPT_MAX and the FZ_MAX evaluations. The nonlinear OPT_MAX load case consistently and significantly resulted in higher $\Vert t\Vert$ than the nonlinear FZ_MAX load case in both models (shape: *p* < 0.001, shape + density: *p* < 0.001). The mean relative difference in $\Vert t\Vert$ between the OPT_MAX and the FZ_MAX load cases was 5.8% ± 8.4% for the shape model and 5.2% ± 3.6% for the shape + density model. Associated ranges are [0.2%, 26.5%] and [0.6%, 12.2%], respectively. Differences in $\Vert t\Vert$ between the shape and the shape + density model were significant for both nonlinear load cases (OPT_MAX: *p* < 0.001, FZ_MAX: *p* < 0.001).

**Fig. 7:**
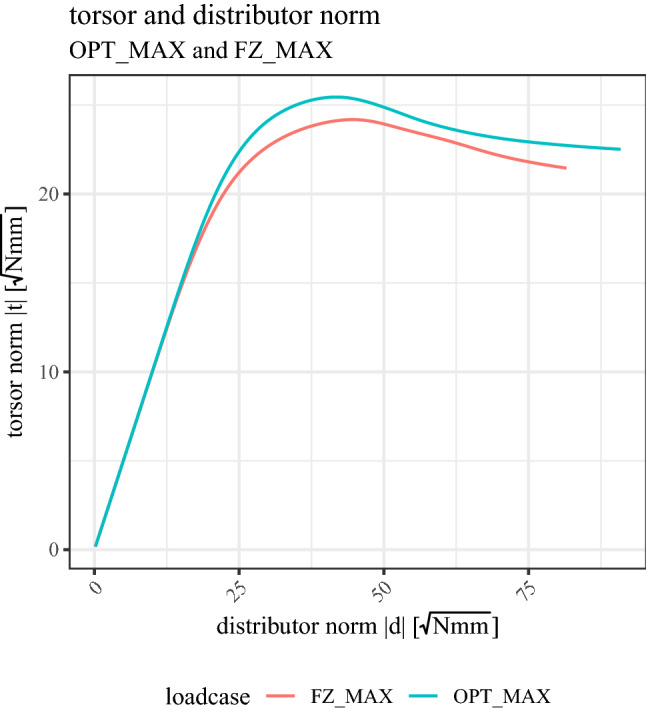
Random example plot of torsor and distributor norm according to formulas 11 and 12. The normalization with the stiffness tensor leads to an initial stiffness equal to 1 for all nonlinear load cases and allows comparison of the ultimate values

An exemplary torsor–distributor norm curve is shown in Fig. 7, and the results of the nonlinear analyses of OPT_MAX and FZ_MAX load case are summarized in Table 1.

## Discussion

The present study aimed to provide a proof of concept for defining personalized loading conditions in hFE analyses of bone strength from in vivo HR-pQCT multi-section reconstructions. The developed optimization approach includes several novelties compared to previous models. First, optimization was based on homogenized finite element models, including six canonical load cases and an optimization strain metric that distinguishes between compression and tension. Furthermore, an approach was developed to compare nonlinear responses of single- and multi-axial load cases by computing a torsor and distributor norm. The bone loading optimization algorithm predicted linear combinations of six linear load cases (OPT) that significantly decreased the OF value, increased the linear energy at the respective optimum, and increased the torsor norm, a surrogate of strength, compared to a standard uniaxial loading boundary condition in the z-direction (FZ) and independent of the model (shape or shape + density).

### Force and moment DOF in OPT load case

**Fig. 8:**
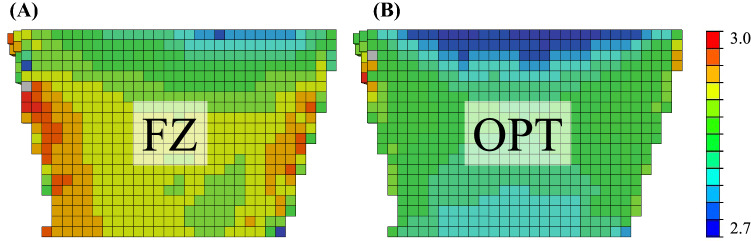
Comparison of OF value maps (hFE mesh cut in the coronal plane) of **A** linear FZ and **B** linear OPT load case

Of the three force DOFs of the linear OPT load case (FX, FY, FZ), a uniaxial force in the z-direction (FZ) was dominant in both models (shape and shape + density). The two other force DOFs (FX, FY) are very small, as these transversal load cases will start building comparatively large shear strains that are immediately penalized by the optimization function. Accordingly, in single load case optimization, ${\alpha }_{i}$ of these two load cases is close to zero and OF values close to 3.0 (maximum, reached for ${\alpha }_{i}=0$). The amplitude of the three moment DOF (MX, MY, MZ) of the linear OPT load case is different in both models. Although energy contributions of the moment DOFs in the linear OPT load case are minimal, the inclusion of moments seems to dissipate inhomogeneities at the periphery seen in the purely axial linear FZ load case and leads to a more homogeneous strain field (Fig. 8). A single DOF model cannot compensate for such inhomogeneities.

### Shape versus shape + density

Including density distribution in the model leads to increased amplitude and sometimes even a sign change in the weights of most linear load cases in the OPT simulation. Even though the inclusion of density distribution increases the inhomogeneity of the element’s bone material properties, the mean OF values decreased for most analyzed models. When the density distribution was added to the shape model, the linear OPT and FZ load case resulted in significantly lower OF values. Nevertheless, only the OPT load case could absorb significantly more energy in the shape + density model than the shape model. This makes sense, as the loading direction in the OPT load case adapts to a new optimum in response to the additional information added by such a density distribution. However, the loading direction of the FZ load case remains unchanged, regardless of the selected model or information. Nonlinear OPT_MAX and FZ_MAX resulted in significantly higher $\Vert t\Vert$ when density distribution was included. Accordingly, the more heterogeneous bone properties ultimately result in a more homogeneous strain distribution, indicated by the lower mean OF values. This suggests that the density distribution in the respective bone segments is not arbitrary but contributes significantly to a more homogeneous strain distribution and the structure’s ability to absorb more energy.

### Qualitative comparison with loading estimations based on the shape of the distal section of radius

**Fig. 9:**
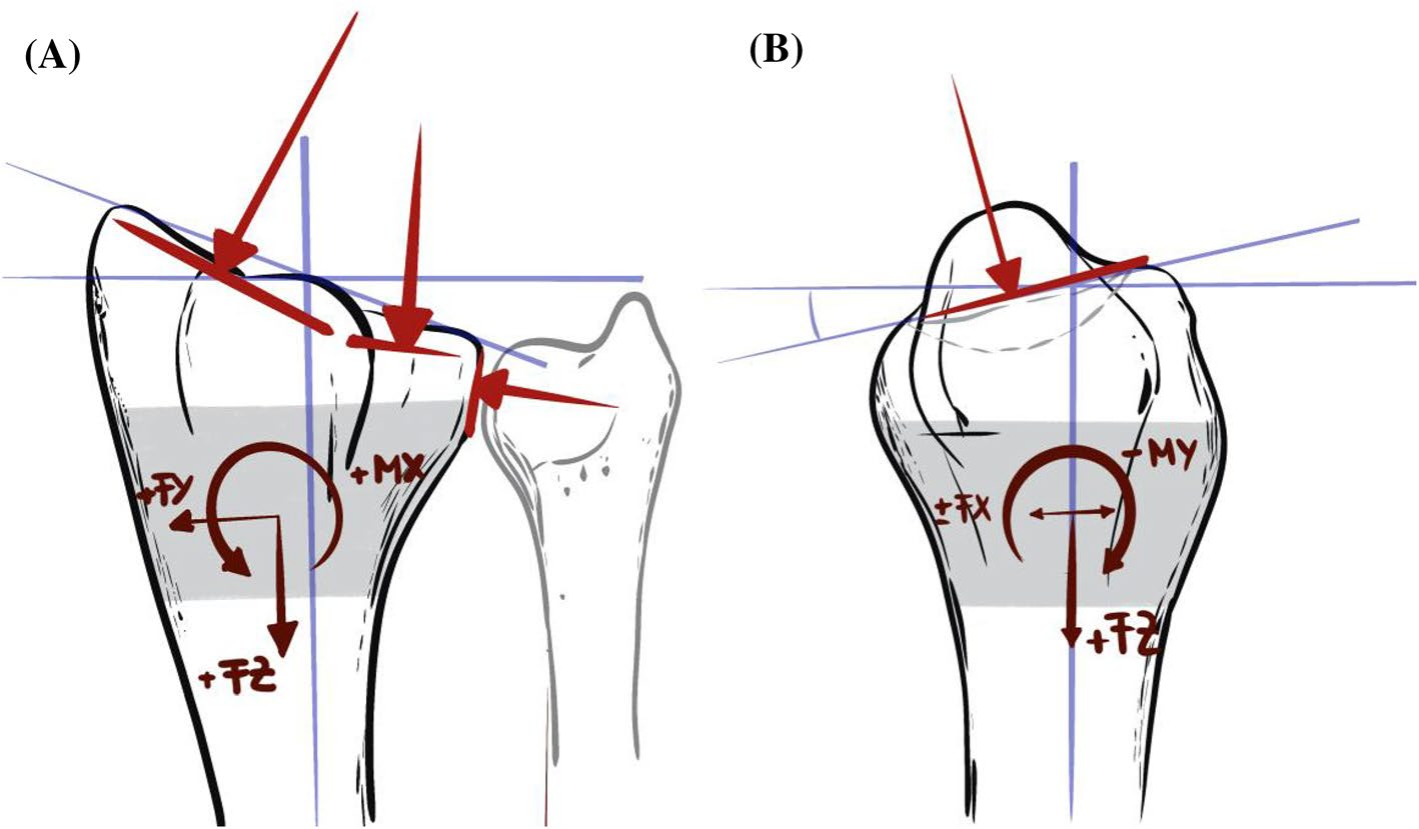
Theoretical analysis of loading directions on a distal segment of a right arm radius resulting from loads transferred from the ulna and the scaphoid-lunate joint surfaces. The distal part of the radius is shown as **A** dorsal view and **B** lateral view. A potential distal subsection of the radius is shown in grey. This does not directly correspond to the double-section samples measured and analyzed in the present study

To set the optimal loading directions into perspective, we studied a nominal shape of the distal part of the human radius. A schematical representation is shown in Fig. 9. We assumed a simplified loading condition of each a force at the scaphoid and lunate joint surface and a force applied from the ulna. Any additional forces from ligaments and muscles were neglected. From the direction of the applied forces and orientation of the joint surfaces, it seems evident that a force in the positive *z*-direction is dominating the resulting forces in the distal subsection. The respective forces in the *x*- and *y*-direction are much smaller. The angulation of the scaphoid and lunate joint surfaces, resulting mainly from the styloid process, will lead to comparatively large moments around the *x*-axis, medium negative moments around the *y*-axis, and smaller torsional moments around the *z*-axis. These observations agree with what we found for our optimal loading directions with the model, including shape and density distribution (shape + density).

### Nonlinear analyses

The computed torsor norms $\Vert t\Vert$ were consistently higher for the nonlinear OPT_MAX load case than for the nonlinear FZ_MAX load case. The respective ranges of relative differences between the two load cases indicate that the impact of the optimization on nonlinear outcomes depends on the individual sample measurement. The positioning of the forearm in the HR-pQCT scanner is prone to angulation errors. We hypothesize that the more the scanner axis (and therefore the subsequent uniaxial loading direction FZ) differs from the axis of the optimal force boundary condition, the more different will nonlinear outcomes such as torsor norms $\Vert t\Vert$ become. If personalized loading directions are insensitive to angulation errors during CT measurements, they might offer a possibility to compensate them and reduce repeatability and reproducibility errors. However, such investigations were not in the scope of the present proof of concept and will maybe even require more advanced models, including microstructural or fabric orientation.

### Limitations

The present study has several limitations that need to be discussed. First, the hFE model we used simplifies several aspects compared to previous ones. The model only differentiates a single bone phase. However, it is known that the material behavior of cortical and trabecular bone is quite different on a macroscopic level. Using a single set of isotropic material properties overestimates the material properties of trabecular bone and underestimates the ones of cortical bone. Furthermore, the current hFE model only maps shape and density distribution but uses an isotropic material behavior and no patient-specific material orientation using a fabric tensor. Even if the bone segment’s high-level response was in good agreement with experimental results, both simplifications mentioned above are expected to degrade local evaluations compared to similar models distinguishing two bone phases with distinct orthotropic material properties. As the optimization procedures rely on local evaluations, a two-phase model including material orientation would be favorable. However, in the case of an orthotropic material description, the optimal isotropic compressive reference strain may not be appropriate anymore and requires the determination of a potentially anisotropic optimal strain tensor maximizing strain energy density.

Second, the present approach includes only six load cases associated with a rigid body motion between the proximal and distal surfaces of the bone section. Under this assumption, these two surfaces deform into planes and do not account for the heterogeneous displacements expected from the density variations under the radio-carpal joint. However, more refined load cases require substantially higher computational resources and may not be suitable for clinical applications.

Furthermore, our optimization function assumes that the adaptation of bone tissue seeks to maximize strain energy density with a constant hydrostatic reference strain state in the case of an isotropic material description. Several studies use different optimization goals, like maximizing strength or stiffness, minimizing density, or eliminating notch stresses and strains. However, bone adaptation and its drivers are still not fully understood, so a strong foundation of evidence is missing. Nevertheless, models such as the presented one give alternative insights into the bone as a mechanical structure and might ultimately help personalize FE models’ loading conditions in a clinical application of bone strength analysis and improve individual predictions and diagnosis. We did not systematically compare the outcomes of our strain-based objective function with previous metrics, as implementing different resolution strategies of the optimization problem would have become necessary, which was beyond the scope of the current proof of concept.

## Conclusion

In conclusion, we presented the proof of concept of an optimization procedure to estimate patient-specific loading boundary conditions for hFE analysis methods. Based on a theoretical analysis of the joint surfaces of the distal radius, the estimated loading directions are plausible. A force in positive *z*-direction dominated the linear OPT load case. This indicates that the standard axial compression boundary conditions used in FE analysis are reasonably close to in vivo loadings. Nevertheless, even with this simplified hFE model, the OPT load case could absorb more than twice the energy of a purely axial load case. Furthermore, we found a large variance of relative differences in the nonlinear strength measure of OPT_MAX and FZ_MAX load cases. This indicates that defining patient-specific load boundary conditions might decrease angulation errors during CT measurements and improve repeatability and reproducibility in FE outcomes such as stiffness and strength. The results encourage extending the present simplified analysis method to include multiple bone phases with fabric anisotropy and applying it to existing datasets to test the hypothesis of improved repeatability due to the compensation of angulation errors during scanning.

## Appendix: Additional material

#### Description and experimental validation of an adapted hFE analysis method

##### Material and methods

###### HR-pQCT measurement, image processing and experimental compression test

Hosseini et al. (2017a) established all data in a previous study. In brief, all radius samples were measured using a second-generation HR-pQCT scanner (XCT2, Scanco Medical AG, Brütisellen, Switzerland) with standard clinical settings at an isotropic voxel size of 60.7 μm. Then, the scanned radius double-sections were dissected and parallelly cut (Exakt bandsaw model 30, Germany). All CT reconstructions were processed according to the clinical standard procedures, including the definition of the periosteal contour and segmentation according to Laib and Rüegsegger (1999). Mechanical compression tests were performed on an experimental setup adopted from Varga et al. (2010). The experimental setup consisted of two steel plates with sandblasted surfaces. The bottom plate (proximal surface of radius segments) was rigidly coupled to a load cell, and the top plate (distal surface of radius segments) was connected to a ball joint with its center of rotation located on the distal surface of the radius segments. The samples were loaded with a quasi-static displacement rate of 5 mm/min (Chevalier et al. 2008) up to a maximum compressive deformation of about 4.6 mm. Displacement of the top plate was measured with an optical capture system (Optotrac, NDI, Canada). From the resulting force–displacement data, experimental stiffness was computed as the maximum slope of a moving linear regression and failure or ultimate load as the maximum recorded reaction force.

###### Mesh generation and material homogenization

The periosteal masks were downscaled by a factor of 12, chosen based on a mesh conversion analysis, and because double-section HR-pQCT measurements contain 336 slices (168 per section), a multiple of 12. The resulting image consists of voxels with an isotropic size of 0.98 mm. Then for each of these downscaled image voxels, we computed the volume fraction ${\phi }_{B}$ that was occupied by the periosteal mask. All downscaled voxels with ${\phi }_{B}>0$ were converted to 8-node hexahedral elements (Abaqus: C3D8) to form the mesh.

All converted FE elements were assigned homogenized material properties. First, the original grey-level reconstruction values were converted to bone mineral density (BMD) values using the BMD calibration equation implemented on the scanner and controlled by daily quality control measurements. The BMD values were then converted to BMD-true bone volume fraction (BV/TV) values (BV/TVd) by dividing BMD by a factor of 1200, as 1200 mgHA/cm^3^ is considered the mineral density of cortical bone (compact bone with a density of 2 g/ccm and a mineral volume fraction of 60%). BMD-true BV/TV values have been shown to depend on image resolution (Varga et al. 2009). Therefore, we decided to linearly calibrate BV/TVd to BV/TV based on the segmentation of 16.5 μm μCT reconstructions, established by Hosseini and colleagues (slope = 0.963, intercept = 3.814%) (Hosseini et al. 2017b).

At the centroid of each FE element, a spherical region with a diameter of 2.482 mm was defined. Then, each element’s bone density ${\rho }_{B}$ was computed as the average of the calibrated grey-level BV/TVd image within the spherical region and the periosteal mask. Each elements’ density ${\rho }_{B}$ was then weighted by the partial volume fraction ${\phi }_{B}$. If an element is only partially occupied by the periosteal mask, then ${\phi }_{B}<1$.

###### Material homogenization

The mechanical behavior of bone tissue was modeled in a simplified manner using an isotropic elastic–plastic constitutive model including damage, adapted from Schwiedrzik and Zysset (2013). The initial material properties before material calibration are based on the trabecular bone properties proposed by Cowin (2001), Daszkiewicz et al. (2017), Panyasantisuk et al. (2015). The initial response is modeled as linear elastic. Then follows yielding and the accumulation of damage and irreversible strains, reducing the stiffness tensor components.

###### Fitting of material properties and validation

Because of significant differences in the hFE methodology compared to previous analysis methods, we had to refit the material properties and validate the present method using the experimental results described above, acquired from Hosseini et al. (2017a).

In the hFE models, the displacements of all nodes at the most proximal surface were fixed in all 6 DOFs. The nodes at the most distal surface were kinematically coupled to a virtual reference node positioned at the distal surface along the central, longitudinal axis of the bone volume. The reference node was then loaded along the 3rd DOF with a uniform axial displacement of up to roughly 2% of strain. Reaction force and displacement of the virtual node along the 3rd DOF were recorded. Stiffness was computed as the initial slope of the resulting force–displacement curve, and strength or ultimate load was defined as the maximum recorded reaction force.

We then compared experimentally measured stiffness and ultimate load to the respective hFE outcomes using linear regression models. The material properties were grouped in elastic and strength/yield properties. Both groups were calibrated with a separate scaling variable (SCA1: elastic properties, SCA2: strength/yield properties, acc. to Table 2) to minimize the root mean squared error (RMSE) between experimental and hFE outcomes.

**Table 2:** Fitted material properties used for hFE analysis method (bold parameters were scaled)

Bone material model			Scaling
**Young’s modulus**	${{\varvec{E}}}_{0}[{\varvec{G}}{\varvec{P}}{\varvec{a}}]$	**18.947**	SCA1
**Shear modulus**	${{\varvec{\mu}}}_{0}[{\varvec{G}}{\varvec{P}}{\varvec{a}}]$	**7.602**	SCA1
Poisson’s ratio	${v}_{0}$[–]	0.2461	
Power for modulus-density relationship	$k [-]$	1.63	
Power for modulus-fabric relationship	$l [-]$	1.1	
**Tensile strength**	${{\varvec{\sigma}}}_{0}^{+}[{\varvec{M}}{\varvec{P}}{\varvec{a}}]$	**127.16**	SCA2
**Compressive strength**	${{\varvec{\sigma}}}_{0}^{-}[{\varvec{M}}{\varvec{P}}{\varvec{a}}]$	**184.73**	SCA2
**Shear strength**	${{\varvec{\tau}}}_{0}[{\varvec{M}}{\varvec{P}}{\varvec{a}}]$	**97.74**	SCA2
Power for strength-density relationship	$P$[–]	1.69	
Power for strength-fabric relationship	$q$[–]	0.98	
Multiaxial interaction coefficient	$\zeta$[–]	0.1876	

##### Results

The results of the material properties calibration for the adapted and simplified hFE analysis method are summarized in Table 2, and results of the linear regression analyses for experimental validation are summarized in Table 3.

**Table 3:** Summary of experimental validation outcomes for stiffness and strength

Parameter	Stiffness	Strength
Slope	0.997	1.001
Intercept	1608 N/mm	1180 N
R^2^	0.889	0.952
RMSE	7281 N/mm	743 N
Lin’s concordance correlation coefficient	0.942	0.922

## References

[CR1] Arias-Moreno AJ, Hosseini HS, Bevers M, Ito K, Zysset P, van Rietbergen B (2019). Validation of distal radius failure load predictions by homogenized- and micro-finite element analyses based on second-generation high-resolution peripheral quantitative CT images. Osteoporos Int.

[CR2] Baumbach SF, Schmidt R, Varga P, Heinz T, Vécsei V, Zysset PK (2011). Where is the distal fracture line location of dorsally displaced distal radius fractures?. J Orthop Res.

[CR3] Beaupré GS, Orr TE, Carter DR (1990). An approach for time-dependent bone modeling and remodeling-application: a preliminary remodeling simulation. J Orthop Res.

[CR4] Burger EH, Klein-Nulend J (1999). Mechanotransduction in bone—role of the lacunocanalicular network. FASEB J.

[CR5] Carter DR, Beaupré GS (2001). Skeletal function and form: mechanobiology of skeletal development, aging, and regeneration.

[CR6] Chevalier Y (2008). A patient-specific finite element methodology to predict damage accumulation in vertebral bodies under axial compression, sagittal flexion and combined loads. Comput Methods Biomech Biomed Engin.

[CR7] Christen P, Van Rietbergen B, Lambers FM, Müller R, Ito K (2012). Bone morphology allows estimation of loading history in a murine model of bone adaptation. Biomech Model Mechanobiol.

[CR8] Christen P, Ito K, Knippels I, Müller R, van Lenthe GH, van Rietbergen B (2013). Subject-specific bone loading estimation in the human distal radius. J Biomech.

[CR9] Christen P, Ito K, dos Santos AA, Müller R, Van Rietbergen B (2013). Validation of a bone loading estimation algorithm for patient-specific bone remodelling simulations. J Biomech.

[CR10] Christen P (2016). Voxel size dependency, reproducibility and sensitivity of an in vivo bone loading estimation algorithm. J R Soc Interface.

[CR11] Cowin SC (2001). Bone mechanics handbook.

[CR12] Cowin SC, Hegedus DH (1976). Bone remodeling I: theory of adaptive elasticity. J Elast.

[CR13] Daszkiewicz K, Maquer G, Zysset PK (2017). The effective elastic properties of human trabecular bone may be approximated using micro-finite element analyses of embedded volume elements. Biomech Model Mechanobiol.

[CR14] Fischer KJ, Jacobs CR, Carter DR (1995). Computational method for determination of bone and joint loads using bone density distributions. J Biomech.

[CR15] Fischer KJ, Jacobs CR, Levenston ME, Carter DR (1996). Different loads can produce similar bone density distributions. Bone.

[CR16] Hegedus DH, Cowin SC (1976). Bone remodeling II: small strain adaptive elasticity. J Elast.

[CR17] Hosseini HS (2017). Fast estimation of Colles’ fracture load of the distal section of the radius by homogenized finite element analysis based on HR-pQCT. Bone.

[CR18] Hosseini HS, Pahr D, van Rietbergen B, Zysset PK (2017). Fast estimation of Colles’ fracture load of the distal section of the radius by homogenized finite element analysis based on HR-pQCT. Bone.

[CR19] Huiskes R, Rulmerman R, Van Lenthe GH, Janssen JD (2000). Effects of mechanical forces on maintenance and adaptation of form in trabecular bone. Nature.

[CR20] Laib A, Rüegsegger P (1999). Comparison of structure extraction methods for in vivo trabecular bone measurements. Comput Med Imaging Graph.

[CR21] Mueller TL (2011). Computational finite element bone mechanics accurately predicts mechanical competence in the human radius of an elderly population. Bone.

[CR22] Panyasantisuk J, Pahr DH, Gross T, Zysset PK (2015). Comparison of mixed and kinematic uniform boundary conditions in homogenized elasticity of femoral trabecular bone using microfinite element analyses. J Biomech Eng.

[CR23] Samelson EJ (2019). Cortical and trabecular bone microarchitecture as an independent predictor of incident fracture risk in older women and men in the Bone Microarchitecture International Consortium (BoMIC): a prospective study. Lancet Diabetes Endocrinol.

[CR24] Schenk D, Mathis A, Lippuner K, Zysset P (2020). In vivo repeatability of homogenized finite element analysis based on multiple HR-pQCT sections for assessment of distal radius and tibia strength. Bone.

[CR25] Schwiedrzik JJ, Zysset PK (2013). An anisotropic elastic-viscoplastic damage model for bone tissue. Biomech Model Mechanobiol.

[CR26] Stuck AK, Schenk D, Zysset P, Bütikofer L, Mathis A, Lippuner K (2020). Reference values and clinical predictors of bone strength for HR-pQCT-based distal radius and tibia strength assessments in women and men. Osteoporos Int.

[CR27] Varga P, Baumbach S, Pahr D, Zysset PK (2009). Validation of an anatomy specific finite element model of Colles’ fracture. J Biomech.

[CR28] Varga P, Pahr DH, Baumbach S, Zysset PK (2010). HR-pQCT based FE analysis of the most distal radius section provides an improved prediction of Colles’ fracture load in vitro. Bone.

[CR29] Varga P, Dall’Ara E, Pahr DH, Pretterklieber M, Zysset PK (2011). Validation of an HR-pQCT-based homogenized finite element approach using mechanical testing of ultra-distal radius sections. Biomech Model Mechanobiol.

